# Supplementary material for: Awareness, knowledge and trust in the Greek authorities towards COVID-19 pandemic: results from the Epirus Health Study cohort
Source: BMC Public Health. 2021 Jun 12;21:1125. doi: 10.1186/s12889-021-11193-x (PMC8196287; doi:10.1186/s12889-021-11193-x)
Supplement: Supplementary file 1 — Additional file 1: Supplementary Figure 1. Categorization of the levels of knowledge regarding the COVID-19 pandemic in Epirus Health Study (EHS). [file 12889_2021_11193_MOESM1_ESM.pdf]

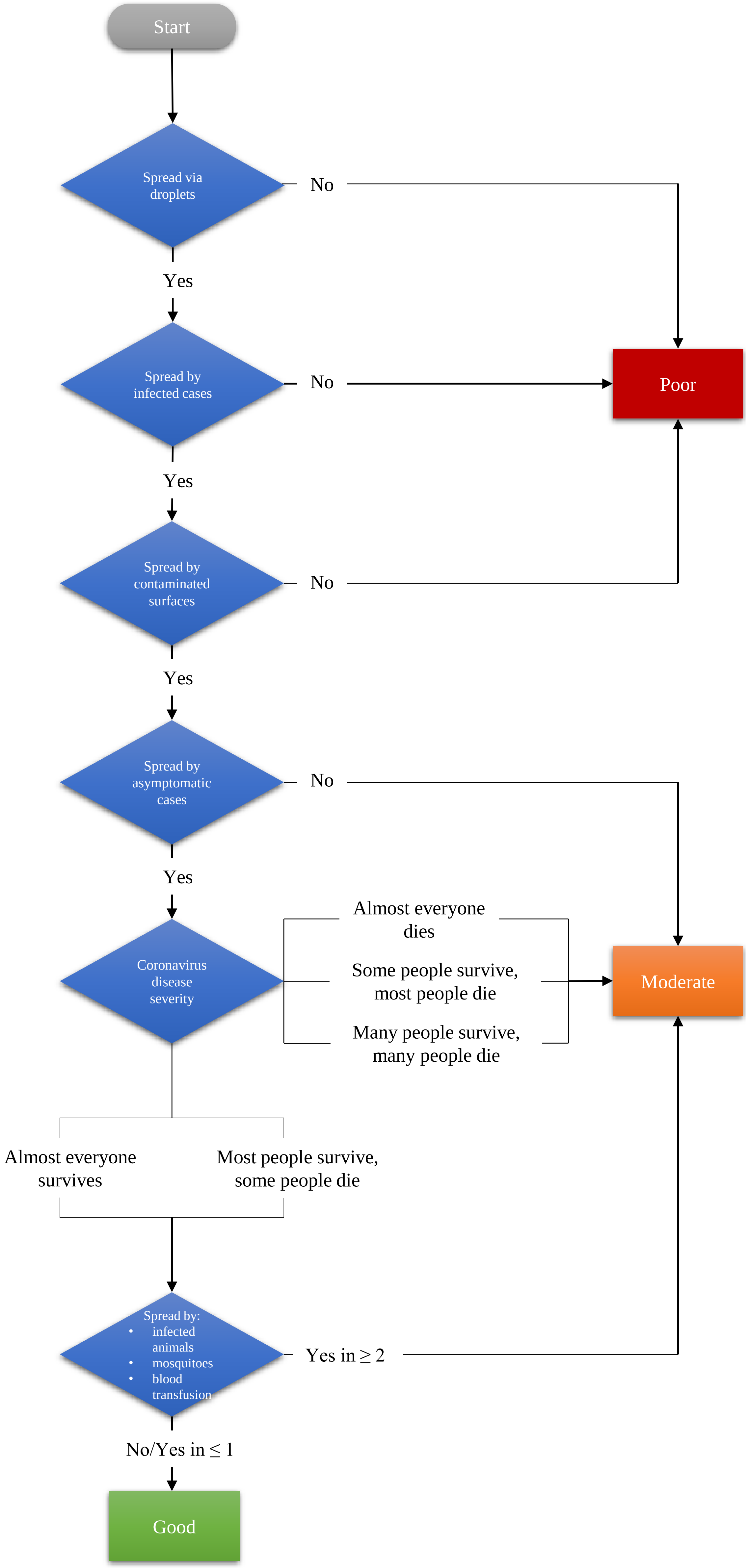

Supplementary Figure 1: Categorization of the levels of knowledge regarding the COVID-19 pandemic in Epirus Health Study (EHS)
